# Shared and targeted barriers to care for skin-related neglected tropical diseases in two endemic communes of Benin: Toward a framework for resource allocation

**DOI:** 10.1016/j.dialog.2026.100350

**Published:** 2026-09-16

**Authors:** Roch Houngnihin, Mélissa Biao, Bernice Gbèbioho, Fréjus Togonou, Daniel Azonchiga

**Affiliations:** Laboratory of Applied Medical Anthropology (LAMA), Department of Sociology-Anthropology, Faculty of Humanities and Social Sciences, University of Abomey-Calavi, Abomey-Calavi, Benin

**Keywords:** Neglected tropical diseases, Buruli ulcer, Mixed methods, Benin, Implementation science, Health equity, Resource allocation

## Abstract

**Purpose:**

Skin-related neglected tropical diseases (skin-NTDs) persist across sub-Saharan Africa despite sustained control. Programme managers allocating scarce resources must decide whether barriers to care call for shared measures applied uniformly across districts or targeted responses tailored to each setting. We characterized the social, economic and cultural barriers to skin-NTD care in two contrasting endemic communes of Benin, tested for each barrier whether it is uniform or locally differentiated, and set out a preliminary framework for resource allocation that follows from this distinction.

**Methods:**

Convergent mixed-methods study in Zagnanado and Allada, combining a household-based survey of 403 persons affected by a skin-NTD and members of their entourage with 60 semi-structured interviews. Each principal barrier was modelled by multivariable logistic regression with multidimensional adjustment for education, urbanicity, household size, occupation and sex, and tested across two sensitivity specifications. Reporting followed STROBE and COREQ.

**Results:**

Treatment cost was reported by 84.6% of participants with no detectable between-commune difference (adjusted odds ratio [aOR] 0.72; *P* = 0.380). Limited access (26.4% versus 66.7%) and lack of infrastructure (10.0% versus 53.0%) were markedly concentrated in Zagnanado and persisted after adjustment (aOR 8.09 and 12.91; both *P* < 0.001), robust across three specifications of socioeconomic adjustment. Qualitative data linked these barriers to inability to pay upfront, peripheral health-system under-resourcing, and a local Fongbe nosology directing patients first to traditional or faith healers.

**Conclusion:**

Cost behaves as a shared, high-burden barrier; structural deficits are locally concentrated. We propose a preliminary framework that classifies each barrier by its burden and between-setting variability, matching shared measures to shared barriers and targeted investment to locally concentrated ones. The framework is derived from two purposively selected communes and has not been externally validated; it is offered as a hypothesis for programme managers to test, not as a ready-to-use instrument.

## Highlights

• Treatment cost was reported by ∼85% of affected participants in both communes.
• Access and infrastructure deficits concentrated in one commune (aOR 8 and 13).
• Local Fongbe nosology channels patients first to traditional or faith healers.
• A proposed framework sorts barriers by burden and between-setting variability.
• Shared measures fit shared barriers; targeted investment fits localized ones.

## 1 Introduction

Social, economic and environmental conditions shape population health more powerfully than biomedical factors alone: the World Health Organization attributes 30% to 55% of health-outcome differences worldwide to social determinants such as education, income, housing, and access to water and sanitation [1]. These determinants bear most heavily on the world's poorest. In sub-Saharan Africa, where a large share of the population lives in poverty and only about half can reach basic health services [2], they create the conditions in which neglected tropical diseases (NTDs) take hold. The persistent and disproportionate burden of NTDs on poor populations is a paradigmatic illustration of what Link and Phelan [3] termed a fundamental cause of health inequality.

Skin-related NTDs (skin-NTDs), notably Buruli ulcer, leprosy, cutaneous leishmaniasis, onchocerciasis, lymphatic filariasis, scabies, yaws and mycetoma [4], cause physical suffering, long-term disability, social stigma and income loss. Around 1.65 billion people require preventive or curative NTD interventions each year, with about 40% of cases in sub-Saharan Africa [5]. In Benin, skin-NTDs persist despite the National Programme for the Control of Leprosy, Buruli Ulcer and Yaws [6], [7]. They particularly affect rural areas of structural poverty, where populations farm, rear livestock and fish in environments conducive to transmission. The communes of Zagnanado and Allada are endemic areas where Buruli ulcer detection and treatment centres (Centres de Détection et de Traitement de l'Ulcère de Buruli, CDTUB) operate as specialized public facilities managing Buruli ulcer, leprosy and other skin-NTDs and serve as the entry point to free protocolized care; limited peripheral infrastructure, persistent traditional beliefs and frequent recourse to traditional medicine nonetheless hinder early detection and adequate management.

Although skin-NTD persistence is a global concern, its drivers are enacted locally, so effective implementation of control programmes requires understanding context-specific social realities. This creates a recurring dilemma for programme managers across West Africa: should scarce resources fund shared measures applied uniformly across districts, or targeted responses tailored to each setting? Answering this requires knowing whether the barriers to care are themselves uniform or locally differentiated, a question seldom tested directly. The literature on access to care offers conceptual maps of how patients are or are not able to reach services [8], [9] and how prospective users come to see themselves as legitimate candidates for those services [10], but these frameworks have rarely been operationalized for the resource-allocation decisions that programme managers actually face. The present study has three aims. First, to characterize the social, economic and cultural barriers to skin-NTD care reported by affected individuals and their families in two contrasting endemic communes of Benin. Second, to test, for each principal barrier, whether it is uniform across the two settings or locally differentiated, using multidimensional socioeconomic adjustment and robustness checks across alternative specifications. Third, to derive from this comparison a preliminary, practice-oriented framework that classifies each barrier by its burden and its between-setting variability and links each type to a distinct intervention logic, illustrated rather than validated here. The study forms part of the DESABEN-NTD project (Social Determinants of Health and Neglected Tropical Diseases).

## 2 Conceptual framework

The analysis is anchored in the WHO framework for the social determinants of health [1], distinguishing structural determinants (the socioeconomic and political mechanisms that generate social stratification) from intermediary determinants (material circumstances, behaviours and the health system). Within this framework we mobilize four complementary perspectives. The fundamental-cause theory [3], [11] explains why socioeconomic position remains a determinant of access even after specific intermediate risks are controlled: socioeconomic resources confer flexible advantages (money, knowledge, power, prestige and beneficial social connections) that can be deployed across whatever proximate barriers a health system happens to present. The access framework of Levesque et al. [8], which conceptualizes accessibility as the fit between the abilities of populations and five dimensions of services (approachability, acceptability, availability, affordability, appropriateness), provides the typology against which we read our reported barriers. The concept of candidacy [10] directs attention to how patients come to identify themselves as eligible for biomedical care. The theory of social representations [12], combined with structural vulnerability [13], accounts for how local nosology and structural position together shape the therapeutic itinerary. The One Health perspective was additionally drawn upon, in a descriptive rather than analytical capacity, to read the occupational and environmental exposure data [14].

Our proposed framework complements rather than replaces these frameworks: existing frameworks identify which barriers exist, whereas we ask, for each barrier, whether it behaves similarly across settings (favouring a shared response) or concentrates locally (favouring a targeted one), thereby supporting practical resource-allocation decisions by programme managers.

## 3 Methods

### 3.1 Study setting

Zagnanado (Zou department) covers 750 km^2^ with approximately 55,061 inhabitants, predominantly Mahi and Yoruba, with Christian and traditional religions prevailing; it has one CDTUB and six districts. Allada (Atlantic department) covers 381 km^2^ with 125,712 inhabitants, predominantly Aizo (83%) and Fon (10%), with a strong presence of the endogenous religion (63.9%) and 12 districts; the Allada CDTUB is a specialized departmental hospital managing Buruli ulcer, leprosy and other skin-NTDs. Zagnanado and Allada were selected purposively as a contrast pair within the same national programme, on three criteria. First, both are well-documented endemic foci for skin-NTDs and host an operational CDTUB, ensuring access to the affected population and permitting protocol harmonization across sites. Second, they differ markedly on the structural determinants most relevant to access (Zagnanado is more rural, more sparsely served and more remote; Allada is denser, peri-urban and closer to the national reference hospital network), providing the empirical contrast needed to test whether barriers are uniform or locally differentiated. Third, the two communes belong to distinct departments (Zou and Atlantic respectively), with different sociolinguistic and religious profiles, which guards against a single-area finding. The pair is therefore designed for analytical contrast rather than statistical representativeness of all endemic districts of Benin.

### 3.2 Study design and sampling

We used a convergent mixed-methods design [15]: the quantitative and qualitative components were conducted in parallel during a single fieldwork phase and analyzed separately, and the two strands were brought together at the interpretation stage. The interview guide was developed from the conceptual themes of the study rather than from the survey results. Integration was therefore carried out after analysis: the qualitative accounts were used to contextualise and account for the quantitative patterns, the four qualitative domains are set against the corresponding survey findings in Table 4, and the two strands are read jointly in Section 5. This cross-sectional, observational study followed the STROBE guidelines. For the quantitative component, sample size was calculated using the Schwartz formula (expected proportion 0.5 for maximum variance, 95% confidence, 5% margin; computed *n* = 384), rounded up to 404. The formula assumes probability sampling; given the recruitment procedure described below, this figure should be read as a target sample size rather than as a guarantee of the corresponding precision. A sampling frame was constructed by enumerating eligible households in CDTUB intervention areas and surrounding villages, using CDTUB registers and household enumeration to identify persons affected by a skin-NTD and members of their entourage. Eligible participants were persons affected by a skin-NTD, whether or not they attended a CDTUB, and members of their entourage. Persons not affected by a skin-NTD and not belonging to the entourage of an affected person, and persons whose condition was unrelated to skin-NTDs, were not included. The target of 404 was allocated between the two communes in proportion to their number of administrative districts (Allada 269, Zagnanado 135). Data collection was carried out by one field team per commune, each working through the enumerated villages and recruiting eligible individuals as they were encountered until its target was reached; recruitment was therefore consecutive within the enumerated frame rather than a probability sample drawn from it. The achieved sample departed from the planned allocation and comprised 220 participants in Allada (54.6%) and 183 in Zagnanado (45.4%), totalling 403 of the 404 targeted. The number of individuals approached and the number who declined to take part were not recorded, so a participation rate cannot be reported. Although the frame was anchored on CDTUB intervention areas, interviews were conducted in a public place of the participant's own choosing rather than in a health facility, as set out in the study information sheet. Because the frame was built from household enumeration and not from CDTUB registers alone, participants who had never reached a CDTUB but lived in its catchment area are also captured; this reduces the likelihood that the sample over-represents patients already linked to biomedical care. For the qualitative component, the sample size was fixed a priori at 60 participants by purposive quotas across three stakeholder categories: 36 rights-holders (affected persons and relatives), 14 responsibility-holders (modern and traditional providers) and 10 duty-bearers (local authorities and programme officials), the last group being included exhaustively. Within the first two quotas, participants were balanced between those exposed and those not exposed to the integrated programme activities. Saturation was monitored during data collection and analysis to verify that these quotas were sufficient, rather than used to determine the sample size. These categories follow the human-rights-based approach used in NTD programming, and the position of providers within it requires comment. Rights-holders are the persons entitled to care and those who support them. Duty-bearers are the actors formally accountable for delivering that care and for allocating the resources it requires. Providers were grouped separately because their position differs from both: public-sector clinicians act as agents of duty-bearing institutions but do not themselves hold the allocation decisions, while traditional and faith healers carry no formal public obligation yet are, in practice, the first point of contact for many patients. We therefore treat providers as responsibility-holders in the sense of day-to-day operational responsibility, and we distinguish biomedical from traditional providers wherever their accounts differ. Sample size was computed for descriptive prevalence estimation and not for the between-commune comparisons; the logistic-regression analyses are therefore secondary. The large observed effect sizes for the structural barriers (adjusted odds ratio approximately 8 and 13) were detected with narrow confidence intervals, whereas any smaller between-commune differential, including in treatment-cost burden, may have been undetectable at this sample size (see Section 5.3).

### 3.3 Data collection

A structured questionnaire was administered via smartphones using Open Data Kit (ODK) Collect, covering sociodemographic characteristics, economic activities, income, knowledge of skin-NTDs and access to care; it was pre-tested with 20 participants in a non-included commune (Abomey-Calavi), and ambiguous items were reworded. Semi-structured interviews were conducted in Fongbe and French, recorded and transcribed, and a direct-observation grid documented living conditions, hygiene practices and environment. Qualitative reporting follows the COREQ checklist.

### 3.4 Variables and statistical analysis

Following recommendations of social epidemiology when comprehensive asset data are not available [16], [17], we adopted a multidimensional approach to socioeconomic position rather than relying on a single composite index. We adjusted simultaneously for four objective dimensions: education (ordinal, four levels: none, primary, secondary, university), urbanicity of residence (ordinal, three levels: rural, semi-urban, urban), a self-reported item for which no operational definition or external reference was supplied to participants, household size (ordinal, five levels) and occupational status (categorical: farmer or fisher as reference; artisan or trader; manual worker; professional; unemployed; other). Sex (male versus female) was also included. Each barrier outcome was derived from a single multiple-response question asking which economic factors influence skin-NTD prevalence in the participant's setting. Participants who answered that economic factors played no part were not routed to this question. Limited access to care, treatment cost and lack of infrastructure were each coded as a binary indicator taking the value one when the corresponding option was selected and zero otherwise; the barriers are therefore reported perceptions of what constrains care in the community, not measures of the individual's own experience of seeking care.

Quantitative data were analyzed in R (version 4.3.3; generalized linear models fitted with the stats package, variance inflation factors computed with the car package, and Hosmer-Lemeshow calibration assessed with the ResourceSelection package). Between-commune associations for categorical outcomes were tested using the Pearson chi-square test with Yates continuity correction for 2 × 2 tables; a two-sided *P* < 0.05 was used. Each principal barrier (limited access, lack of infrastructure, treatment cost) was modelled by multivariable binary logistic regression with the adjustment described above; adjusted odds ratios (aOR) are reported with their 95% confidence intervals. Confidence intervals are Wald intervals. The full model output, including all occupational-status coefficients, is provided in Table S3.

Qualitative data underwent thematic analysis. Transcripts were imported into NVivo and coded inductively in two stages: open coding produced an initial coding list, which was then organized into a four-domain thematic framework (economic barriers; structural barriers in the health system; nosological and representational barriers; proposed strategies). Coding was conducted by two members of the team, with discrepancies resolved by discussion. Triangulation across the three respondent categories (rights-holders, responsibility-holders, duty-bearers) was used to check the consistency of thematic content. Excerpts presented below were originally collected in French or Fongbe and translated into English by the research team; the original transcripts are available from the corresponding author. To prevent re-identification, excerpts are reported with broad descriptors only (commune, role, ten-year age band) and no village name, surname or distinguishing physical attribute. Data collection continued until thematic saturation, no new codes emerging from additional interviews, was judged to have been reached in each respondent group, and the final interviews confirmed existing themes. The two coders' independent, pre-consensus code assignments were not retained, so a formal inter-rater agreement coefficient could not be computed; coding consistency instead rested on double coding with consensus resolution and on triangulation across the three respondent groups.

### 3.5 Reflexivity and positionality

Interviews were conducted by trained social-science interviewers who were not involved in the participants' clinical care, in the participants' own language (Fongbe) and, in a public place of their own choosing rather than in a health facility, in order to reduce the power asymmetry that a clinical setting imposes on people affected by stigmatising conditions. Interviewers introduced themselves as researchers, emphasized that participation was voluntary and would not affect access to CDTUB care, and invited participants to decline or interrupt at any point. The team debriefed after each fieldwork day to examine how their own positions as educated, urban researchers might shape rapport and interpretation, and this reflexive discussion informed the inductive coding.

### 3.6 Ethics

The study was approved by the National Ethics Committee for Health Research (CNERS) of Benin, the national body designated by the Ministry of Health for the ethical review of health research (reference CNERS 002/2025). Approval was granted in February 2025, before the start of data collection in May 2025. The objectives of the study, the voluntary nature of participation and the right to decline or to withdraw at any time without any consequence for access to CDTUB care were explained to each participant in their own language before enrolment. Written informed consent was obtained from every participant; for participants unable to read, the information sheet and the consent form were read aloud and explained to them, and consent was recorded by thumbprint. The same approval, information sheet and consent procedure covered both the survey and the semi-structured interviews. The information sheet, addressed to affected persons and their entourage, to biomedical and traditional providers and to local and programme authorities, stated that participants' names would be replaced by an identifier and that the findings could be published in scientific journals and at conferences. No minimum age was set as an eligibility criterion, and three participants were aged under 18 years (13 to 14 years). For each of these participants, written informed consent was obtained from a parent or guardian, and the participant gave oral assent before the interview; the study documentation did not include a separate age-adapted assent form. All data were de-identified before analysis.

## 4 Results

### 4.1 Sociodemographic profile

The 403 participants were almost evenly split between women (50.6%) and men (49.4%); 76.9% were aged 25 to 49 years. Christianity predominated (61.8%), followed by endogenous religions (29.5%); the Fon group dominated (82.4%). Half lived in rural areas (50.1%) and education was generally low (38.5% with no formal schooling; Table 1). A higher proportion of participants in Zagnanado than in Allada described their place of residence as urban (19.7% versus 2.7%). Because the item was self-reported against no fixed reference, respondents necessarily situated themselves relative to their own surroundings, so the three categories are not comparable between communes on an absolute scale and should be read as a within-commune gradient. The characterisation of Zagnanado as the more remote setting (Section 3.1) rests on its distance from the national referral hospital network and on the density of peripheral health services, not on this variable. Artisans and traders formed the largest occupational group (41.2%), ahead of farmers, livestock-rearers and fishers (22.8%), other occupations (18.4%), professionals (8.2%), participants without employment (5.2%) and manual workers (4.2%). The distribution of occupations differed markedly between the two communes (chi-square = 56.07; df = 5; *P* < 0.001): farming and unemployment were more frequent in Zagnanado, whereas professional occupations were concentrated in Allada (31 versus 2 participants). Participants worked predominantly in the informal sector with irregular incomes, and their activities expose them daily to humid, unsanitary environments that are reservoirs of skin-NTD pathogens; injuries from farming tools provide entry points for infection.

**Table 1:** Sociodemographic characteristics of participants, by commune (N = 403).

Characteristic	Allada (*n* = 220)	Zagnanado (*n* = 183)	Total (*n* = 403)	P
Sex: female	111 (50.5)	93 (50.8)	204 (50.6)	1.00
Sex: male	109 (49.5)	90 (49.2)	199 (49.4)	
Age < 25	13 (5.9)	26 (14.2)	39 (9.7)	0.019
Age 25–49	176 (80.0)	134 (73.2)	310 (76.9)	
Age > 49	31 (14.1)	23 (12.6)	54 (13.4)	
Christian	139 (63.2)	110 (60.1)	249 (61.8)	0.002
Endogenous	67 (30.5)	52 (28.4)	119 (29.5)	
Muslim	6 (2.7)	0 (0.0)	6 (1.5)	
Not stated	8 (3.6)	21 (11.5)	29 (7.2)	
Education: none	73 (33.2)	82 (44.8)	155 (38.5)	0.013
Primary	57 (25.9)	54 (29.5)	111 (27.5)	
Secondary	79 (35.9)	41 (22.4)	120 (29.8)	
University	11 (5.0)	6 (3.3)	17 (4.2)	
Residence: rural	125 (56.8)	77 (42.1)	202 (50.1)	<0.001
Semi-urban	89 (40.5)	70 (38.3)	159 (39.5)	
Urban	6 (2.7)	36 (19.7)	42 (10.4)	

Source: DESABEN-NTD survey, May 2025.

### 4.2 Barriers to care: Between-commune comparison

Treatment cost was reported by 341 of the 403 participants (84.6%), with no significant difference between communes (85.9% in Allada versus 83.1% in Zagnanado; chi-square = 0.42; df = 1; *P* = 0.515). It was the most frequently reported barrier in both settings (Table 2). Limited access to care (26.4% versus 66.7%; chi-square = 64.04; *P* < 0.001) and lack of infrastructure (10.0% versus 53.0%; chi-square = 86.73; *P* < 0.001) differed highly significantly, with the higher burden in Zagnanado (Fig. 1).

**Table 2:** Barriers to care by commune, with chi-square tests.

Barrier	Allada n (%)	Zagnanado n (%)	χ^2^ (df = 1)	P
Treatment cost	189 (85.9)	152 (83.1)	0.42	0.515
Limited access to care	58 (26.4)	122 (66.7)	64.04	< 0.001
Lack of infrastructure	22 (10.0)	97 (53.0)	86.73	< 0.001

Pearson chi-square, Yates correction. Multiple responses; percentages calculated within commune on all participants (Allada n = 220; Zagnanado n = 183). Source: DESABEN-NTD survey, May 2025.

**Fig. 1:**
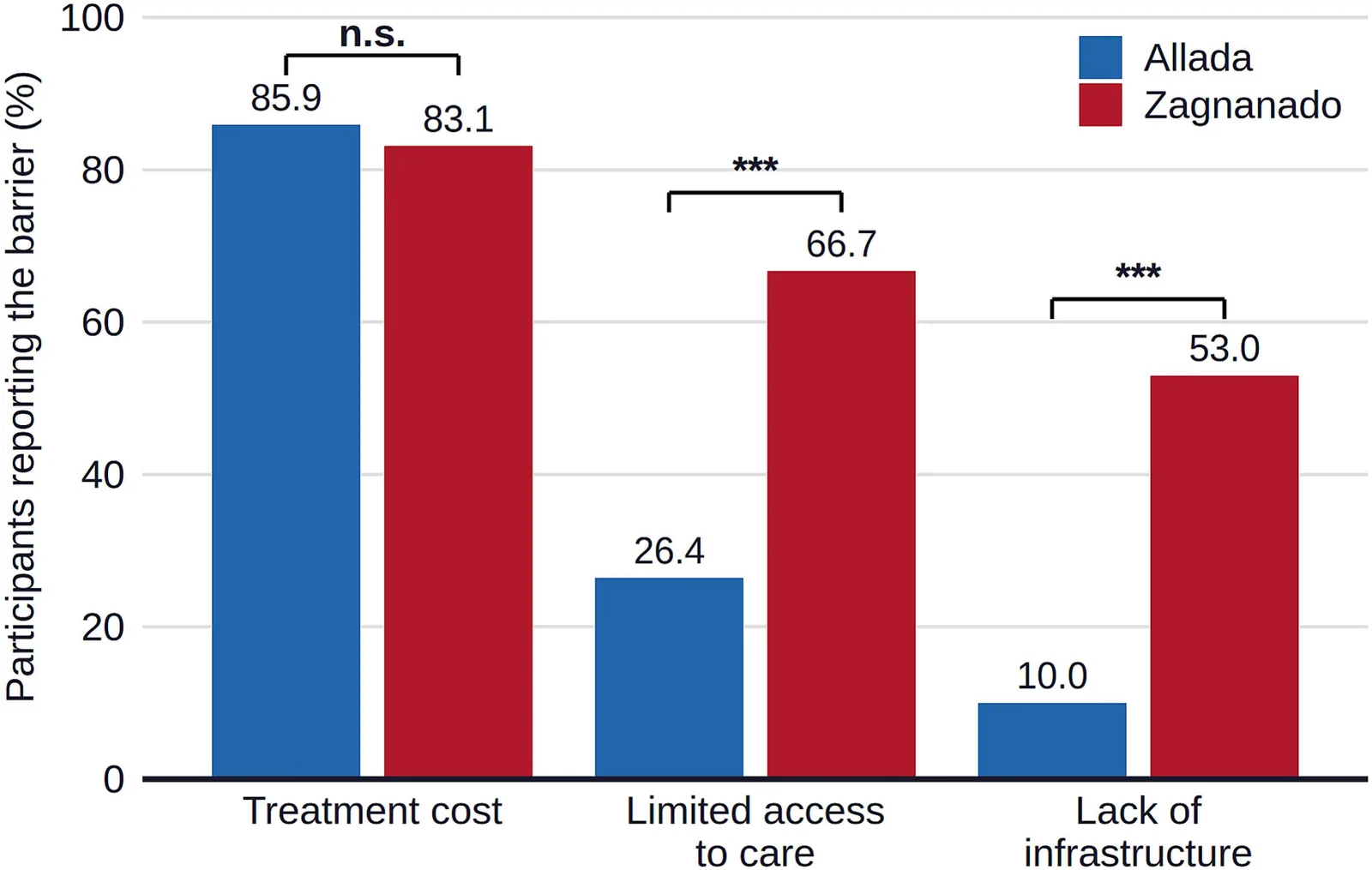
Barriers to care differ structurally between communes. The financial barrier (treatment cost) is similar across communes, whereas limited access and lack of infrastructure are strongly concentrated in Zagnanado. Source: DESABEN-NTD survey, May 2025.

Qualitative accounts grounded these economic barriers in the routine financial impossibility of upfront payment for patients living on irregular informal-sector income:

*“Concerning this question, the problem is that if your relatives or family have no money, you cannot even think of going to the hospital. There, they will talk about money, and without money you can do nothing.” Affected person, Zagnanado, 30–39 years.*

Distance and the cost of transport compound this barrier in the more isolated commune:

*“I cannot walk all the way to Allada where the wounds are treated. Even if you have a motorbike, you still need fuel, you need someone to drive you. And we cannot even manage to eat, so how could I afford that?” Affected person, Zagnanado.*

### 4.3 Multivariable regression with multidimensional adjustment

In multivariable logistic regression, the between-commune contrasts for the structural barriers persisted and indeed strengthened after adjustment for sex, education, urbanicity, household size and occupational status (Table 3). Compared with Allada, residence in Zagnanado was strongly associated with limited access to care (aOR 8.09; 95% CI 4.72 to 13.86; *P* < 0.001) and lack of infrastructure (aOR 12.91; 95% CI 6.71 to 24.81; *P* < 0.001). For treatment cost, no independent commune effect was detectable (aOR 0.72; 95% CI 0.35 to 1.50; *P* = 0.380). The absence of a detectable difference is consistent with treatment cost being a constraint of similar magnitude in both settings, although we cannot rule out a small effect that the study was underpowered to detect. The adjusted results indicate that the concentration of structural barriers in Zagnanado is not an artefact of the differing sociodemographic composition of the two communes. Hosmer-Lemeshow tests with ten groups indicated adequate model fit for all three outcomes (*P* = 0.059, 0.489 and 0.232 for access, infrastructure and cost respectively); the test is, however, sensitive to the number of groups chosen: across 5 to 15 groups the *P* value ranged from 0.02 to 0.45 for limited access, from 0.12 to 0.87 for lack of infrastructure and from 0.01 to 0.38 for treatment cost, so these statistics should be read as indicative rather than as a formal test of fit. Variance inflation factors were all below 10 (maximum 2.07). Education, urbanicity and household size were entered as ordinal variables scored linearly, which preserves their ordering while spending a single degree of freedom each. We checked this assumption by refitting each model with the three variables entered as unordered categories: the linear specification was not rejected for any outcome (likelihood-ratio test, 6 degrees of freedom: *P* = 0.451 for limited access, 0.227 for lack of infrastructure and 0.083 for treatment cost), and the commune effect was essentially unchanged (aOR 8.24, 14.96 and 0.78 respectively). The treatment-cost inference rests on the absence of a large adjusted commune effect, corroborated by the qualitative data. Seventeen of the 403 participants (4.2%) answered that economic factors did not influence skin-NTD prevalence and were therefore not routed to the barrier items. They are counted as not reporting each barrier in Table 2 and are excluded from the regression models; their distribution did not differ between communes (Allada 11/220 versus Zagnanado 6/183; chi-square = 0.37, *P* = 0.54). Excluded and included participants were similar in sex, urbanicity, household size, occupation and age, although educational attainment differed modestly (*P* = 0.02) (Table S2); given the small number excluded, the complete-case analysis is unlikely to introduce material selection bias. The flow of participants is summarized in Fig. S1.

Two sensitivity analyses gave concordant results (Table S4). Replacing the multidimensional adjustment with a single composite SES index from principal component analysis (variance explained by PC1 = 42.4%; loadings: education 0.71, urbanicity 0.61, inverted household size 0.36) yielded commune effects of aOR 5.72 (access), 10.38 (infrastructure) and 0.68 (cost). Using the original enumerator-assigned three-level SES variable instead gave aOR 5.83, 10.74 and 0.68 respectively. The structural-barrier contrasts are therefore robust across three different specifications of socioeconomic adjustment.

**Table 3:** Multivariable logistic regression of barriers to care, principal model (N = 386).

Predictor	aOR	95% CI	P
Outcome: limited access to care (Hosmer-Lemeshow P = 0.059)			
Commune (Zagnanado vs Allada)	8.09	4.72 to 13.86	< 0.001
Sex (male vs female)	0.78	0.46 to 1.33	0.363
Education (per level)	0.80	0.59 to 1.08	0.146
Urbanicity (per level)	1.04	0.71 to 1.52	0.833
Household size (per level)	0.89	0.70 to 1.13	0.324
Outcome: lack of infrastructure (Hosmer-Lemeshow P = 0.489)			
Commune (Zagnanado vs Allada)	12.91	6.71 to 24.81	< 0.001
Sex (male vs female)	1.44	0.80 to 2.59	0.220
Education (per level)	1.02	0.74 to 1.42	0.898
Urbanicity (per level)	1.15	0.77 to 1.71	0.511
Household size (per level)	1.27	0.97 to 1.66	0.081
Outcome: treatment cost (Hosmer-Lemeshow P = 0.232)			
Commune (Zagnanado vs Allada)	0.72	0.35 to 1.50	0.380
Sex (male vs female)	0.99	0.48 to 2.05	0.988
Education (per level)	0.82	0.55 to 1.21	0.315
Urbanicity (per level)	0.63	0.38 to 1.05	0.076
Household size (per level)	1.18	0.84 to 1.66	0.346

aOR, adjusted odds ratio; CI, confidence interval. All models additionally adjusted for occupational status (six categories, farmer/fisher as reference). Reference categories: Allada and female. Source: DESABEN-NTD survey, May 2025.

Qualitative interviews with health workers in the structurally constrained commune linked the access and infrastructure gaps to chronic under-resourcing of peripheral activities:

*“We end up waiting three months or even more before going to the endemic villages, for lack of resources. But if resources were available, we could be in the field every month for awareness and early detection. That would also allow us to transport patients to the CDTUB.” Modern health worker, Allada, 40–49 years.*

Even when participants did initiate biomedical care for a child or relative, peripheral health centres often did not recognize the lesion as a skin-NTD, prolonging the diagnostic itinerary:

*“When my daughter had the spot that was oozing pus, I took her to the health centre in our village. The staff there began giving her care, but as the days went by the wound was not healing, it kept getting worse.” Carer of a patient, CDTUB Zagnanado.*

Many patients first consulted a traditional healer, often paying repeatedly before reaching the CDTUB:

*“The traditional healer reassured us that he treats every kind of wound, whatever its severity, until full healing. But it did not work, otherwise you would not be seeing me here at the CDTUB. We spent a lot of money there without any result, then we came here.” Carer of a patient, CDTUB Allada.*

The 60 interviews are summarized along four thematic domains in Table 4.

**Table 4:** Thematic framework from 60 semi-structured interviews*.*

Domain	Recurrent codes	Triangulation across respondent groups
Economic barriers	Inability to pay upfront; transport costs; lost wages; preference for healer credit systems	Convergent: cited by rights-holders, responsibility-holders and duty-bearers
Structural barriers in health system	Stock-outs of medicines; delayed outreach visits; distance to nearest CDTUB; limited staff	Convergent for Zagnanado; less salient in Allada
Nosological / representational barriers	Fongbe terms for skin lesions (akpa, noudido, wevi kpa, akpa djomakou, azon vo); spiritual aetiology; first recourse to diviner or pastor	Convergent across groups; affirmed by traditional healers themselves
Proposed strategies	Cost removal; outreach decentralization; integration of traditional healers; community education	Priorities differ by commune (financial in Allada, infrastructure in Zagnanado)

Coding by two researchers, with discrepancies resolved by discussion. Thematic saturation was judged to have been reached in each respondent group. Source: DESABEN-NTD qualitative component, 2025.

### 4.4 Knowledge and local representations of skin-NTDs

A large majority (98.5%) reported having heard of NTDs. When shown a predefined list, participants most often selected diseases targeted by mass-treatment campaigns. The predefined list did not include Buruli ulcer or leprosy as named options; the closest category, non-tuberculous mycobacterioses, was selected by 17.4% of participants, and yaws by 7.2% (Table 5). Because the list also included conditions that are not endemic in Benin, responses should be read as recognition of disease names rather than accurate local knowledge. Participants attributed skin diseases mainly to inadequate hygiene (75.9%), diet and skin care (68.0%) and external risk factors (61.3%). This measure captured prompted recognition of disease names from a predefined list, not free recall, recognition of clinical signs, or knowledge of causes, transmission or care pathways, and therefore over-states genuine knowledge. Buruli ulcer was volunteered in the free-text field by three participants (0.7%), despite a specialist CDTUB operating in each commune. This near-absence of the biomedical label is best read alongside the qualitative data, in which the same lesions are named and discussed fluently in Fongbe (akpa and its variants): the label fails to attach to a well-populated local nosology, rather than the disease itself being unknown in the community.

**Table 5:** NTDs recognized from a predefined list, by commune (multiple responses). Rows follow the response options as worded in the questionnaire; the list did not include Buruli ulcer or leprosy as named items.

NTD	Allada n (%)	Zagnanado n (%)	P
Lymphatic filariasis	125 (56.8)	90 (49.2)	0.153
Onchocerciasis	124 (56.4)	51 (27.9)	<0.001
Sleeping sickness	109 (49.5)	57 (31.1)	<0.001
Leishmaniasis	55 (25.0)	57 (31.1)	0.208
Non-tuberculous mycobacterioses	6 (2.7)	64 (35.0)	<0.001
Chagas disease	7 (3.2)	69 (37.7)	<0.001
Yaws	14 (6.4)	15 (8.2)	0.606
Trichinosis	16 (7.3)	25 (13.7)	0.052
Schistosomiasis	15 (6.8)	31 (16.9)	0.002
Lymphatic filariasis (second list entry)	13 (5.9)	49 (26.8)	<0.001
Rhabdomyolysis	1 (0.5)	7 (3.8)	0.040
Dracunculiasis	3 (1.4)	8 (4.4)	0.124
Other	76 (34.5)	4 (2.2)	<0.001

Source: DESABEN-NTD survey, May 2025.

The qualitative material explains how these representations translate into delay. Where illness is interpreted as a cast spell rather than an infection, the first responder is logically a diviner or pastor, not a clinic:

*“When I wake up in the morning with a swollen foot and a large abscess, when the day before there was nothing, it is astonishing. I wonder whether this is a spell, whether it is mystical. So the first thing is to call my pastor or a healer to find out what is happening.” Affected person, Allada.*

Leprosy in particular is interpreted as a sanction for transgression, with implications that travel along family lines:

*“Leprosy is a disease of malediction. When you steal from a sown field, one of the sanctions is the curse of leprosy that enters your family across several generations.” Community leader, Allada, 60–69 years.*

Fears of biomedical care, transmitted within communities, can deter even patients who recognize they are seriously ill:

*“While my foot was swelling, the neighbours kept saying that if I went to the hospital they would amputate it, so I categorically refused to go. (...) I was afraid they would cut off my leg, and others said it would probably be a passing illness.” Affected person.*

The local nosology is elaborate: Buruli ulcer is rendered in Fongbe as akpa (a generic wound) but also, depending on perceived cause, as noudido (the casting of a spell), wevi kpa (a wound caused by bacteria or insects) and akpa djomakou (a wound that does not heal); leprosy is azon vo. The coexistence of naturalistic and personalistic terms for the same lesion shows that a single patient can hold competing explanatory models, each pointing toward a different point of entry into care, an instance of what Dixon-Woods et al. [10] describe as the negotiation through which patients identify themselves as legitimate candidates for biomedical services.

### 4.5 Living conditions, occupational exposure and proposed strategies

Occupational and environmental exposure was a recurring theme. Farming injuries were a frequent point of entry:

*“I was in the field hoeing when the hoe wounded my foot. The next day my foot swelled and I started feeling feverish. When we finally went to the hospital, after pus had begun coming out, the staff diagnosed Buruli ulcer.” Affected person, Zagnanado, 30–39 years.*

Shared water sources concentrate multiple risks in a single place:

*“In an endemic village you see a river, a lake, a streambed, and that is the only place where people fetch water for drinking, washing clothes, cleaning, cooking, bathing. Then they go to the same spot to wash the patient's wound, or for the morning toilet.” Modern health worker, Zagnanado, 50–59 years.*

Living conditions differed between communes: precarious sanitary conditions dominated in Zagnanado (71.6%), whereas vector exposure prevailed in Allada (60.9%). Access to drinking water was limited, with frequently broken infrastructure; in some localities households travel up to 7 km for water. Overall, 91.3% of participants reported skin-NTD persistence in their community. Proposed strategies differed significantly by commune: financial support was the priority in Allada (62.3% versus 23.0%; *P* < 0.001), whereas Zagnanado emphasized infrastructure (39.3% versus 16.4%; *P* < 0.001) and human resources (32.2% versus 17.7%; *P* = 0.001). Integrating traditional medicine was strongly supported, significantly more in Allada (86.8% versus 69.4%; *P* < 0.001). Water and sanitation and hygiene education did not differ significantly, indicating shared cross-commune priorities. Local authorities themselves articulated practical, low-cost adjustments to water-supply pricing that could improve access:

*“Instead of paying 35 CFA francs for a 25-litre can or 50 CFA francs for a large basin, it would be better to pay a 200 CFA franc monthly flat fee per household.” Local elected official, Zagnanado, 40–49 years.*

## 5 Discussion

Affected persons present a multidimensional vulnerability profile: the predominance of farming, low education and rural residence confirms that skin-NTDs mainly affect deprived rural populations [18], and the informal nature of economic activities limits the capacity to access care [19], [20]. The Beninese specificity lies in the importance of the informal sector and the double vulnerability linked to aquatic activities (fishing, rice-growing, raffia collection) that expose populations to hydromorphic environments where *Mycobacterium ulcerans* develops.

The central empirical observation is that the barriers do not behave alike across the two settings. Limited access and lack of infrastructure were sharply concentrated in Zagnanado, with aOR 8.09 and 12.91, and these contrasts were essentially unchanged across three different specifications of socioeconomic adjustment. Treatment cost, by contrast, was reported by most participants in both settings and showed no detectable commune effect in any specification. Statistically, our data do not detect a difference in the cost burden between the two communes; considered together with the qualitative material, in which inability to pay upfront and reliance on healer credit systems were named by rights-holders, responsibility-holders and duty-bearers alike in both communes, it is consistent with treatment cost operating as a shared constraint. The magnitude is consistent with documented catastrophic costs of Buruli ulcer treatment in Ghana and Nigeria, where families spend 20% to 40% of annual income on care [21], [22].

### 5.1 A preliminary framework for classifying barriers

To translate this contrast into a usable decision aid, we propose that programmes organize barriers along two dimensions: burden (the proportion of affected participants reporting the barrier; high when reported by a majority, operationally more than 50%, in at least one setting) and between-setting variability (assessed by a between-setting effect that survives multivariable adjustment). The 50% cut-off is a pragmatic convention rather than a validated threshold; the classification here is not sensitive to the exact cut-off, since the high-burden barriers exceed 50% by a wide margin while the others fall well below it. Two cautions apply to the variability dimension. First, each barrier is placed in a cell on the basis of a point estimate whose confidence interval is wide (Table 3), so the placement inherits the uncertainty of the underlying estimate rather than resolving it. Second, a between-commune effect that does not reach significance, as for treatment cost, means only that a differential was not detected at this sample size; it is not evidence that the barrier is genuinely uniform. No equivalence testing was performed, and the “uniform” column should be read as “no differential detected” rather than as “shown to be the same”. Crossing these two dimensions yields four types, each implying a distinct intervention logic (Fig. 2). Barriers that are high-burden and shared across settings call for shared interventions (national or supra-local); barriers that are high-burden but locally concentrated call for targeted investment; the two low-burden cells warrant routine monitoring or local surveillance. Applied to our data, treatment cost is high-burden and not differentially distributed between the two communes, fitting the first cell (Fig. 2, upper left); limited access and lack of infrastructure are high-burden and locally concentrated, fitting the second (Fig. 2, upper right). We do not interpret a non-significant between-commune contrast as proof of universality; rather, the absence of a detectable differential, in the presence of a high overall burden, signals that an intervention designed for the shared pattern is a defensible default. The intended value of the tool lies in the analytical move it encourages, namely testing each barrier for between-setting variability before choosing between a shared and a targeted response, rather than in our specific figures. This invariance is confirmed by a formal check across burden cut-offs of 40, 50 and 60%, over which every barrier retains its quadrant (Table S5).

**Fig. 2:**
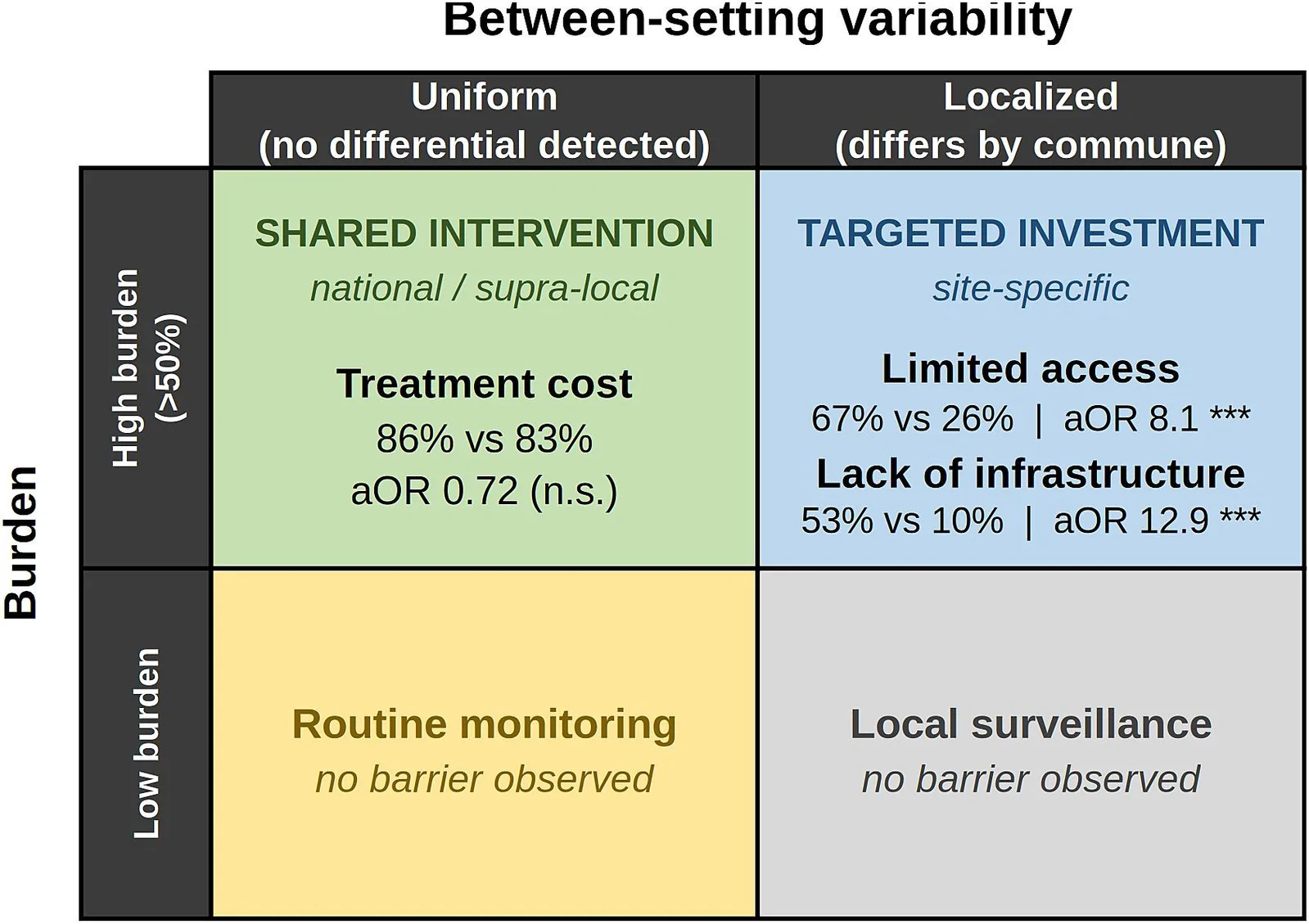
Proposed framework for classifying barriers to skin-NTD care. Each barrier is classified by burden (percentage of affected participants reporting it; high when above 50% in at least one commune) and by between-setting variability (a commune effect surviving multivariable adjustment). High-burden barriers for which no between-commune differential was detected (treatment cost) call for shared interventions; high-burden, locally concentrated barriers (limited access, lack of infrastructure) call for targeted investment. The classification is invariant to burden cut-offs of 40 to 60% (Table S5).

This decision logic is designed to be used alongside existing access frameworks [8], [10]. Levesque et al. describe what barriers exist; we propose a way of deciding, for each, where the intervention should be targeted. Read against Link and Phelan's fundamental-cause theory [3], the salience of treatment cost as a high-burden barrier across both settings is consistent with what is predicted when socioeconomic resources are themselves uneven across the population: the proximate barrier (cost) is the local manifestation of a more fundamental constraint. The dissociation between a rich local nosology and the biomedical label, and the consequent first recourse to traditional or faith healers, has been described in Cameroon and Côte d'Ivoire as well as Benin [23], indicating that the cultural barrier is regionally recurrent even as its content varies by setting. That the biomedical term for Buruli ulcer was volunteered by only three participants, despite both CDTUB specializing in its management, points to the same dissociation between the clinical label and a rich local naming system. Farming, fishing and market-gardening expose populations to humid, marshy environments conducive to skin-NTD pathogens, corroborating findings that climatic and socioeconomic factors shape cutaneous leishmaniasis dynamics [24]. The One Health approach proves relevant: populations rearing livestock and fishing in hydromorphic zones are doubly exposed, and shared water sources create cross-contamination cycles.

Read through the framework of Levesque et al., the three barriers load onto distinct dimensions of access: treatment cost onto affordability, limited access onto availability and accommodation, and lack of infrastructure onto approachability and availability. Their differing geography maps directly onto our matrix, since affordability behaves as a uniform, high-burden constraint whereas the availability-side barriers concentrate locally.

### 5.2 Implications for practice

These findings point toward programmatic priorities, with the caveat that a cross-sectional design identifies associations and perceptions rather than causes, and cannot show that any of the measures discussed below would improve outcomes. Because treatment cost was reported at a similarly high level in both communes, the logical response is a measure applied across endemic settings rather than confined to one: removing point-of-care costs for skin-NTD diagnosis and treatment, or extending financial-protection schemes to affected households. The Beninese context is favourable in principle, given the ongoing rollout of the Insurance for Strengthening Human Capital (Assurance pour le Renforcement du Capital Humain, ARCH) scheme, which could in principle cover skin-NTD treatment for the poorest. The practical question is one of political economy: whether NTD treatment, classically under-funded because the diseases affect politically peripheral populations [25], will compete successfully for ARCH coverage. Because access and infrastructure deficits are locally concentrated, they call for targeted investment where they cluster: decentralized detection points, transport or outreach support, and strengthening of peripheral facilities and the workforce. These too face a political-economy challenge, since infrastructural investment in remote rural districts depends on a sustained political coalition and on the predictability of donor commitments to NTD control.

The strong endorsement of integrating traditional medicine, significantly higher in Allada, supports structured collaboration with traditional and religious providers, who are frequently the first point of contact. Training them to recognize early lesions and refer, rather than treating them as competitors, is a plausible route to earlier presentation whose effectiveness for skin NTDs in Benin remains unproven; in our interviews, traditional healers themselves spoke openly of treatments that had failed and of patients who reached the CDTUB after long delays. Community-based models that integrate local groups to improve awareness and reduce stigma [26], together with gender-sensitive and inclusive design [27], provide a delivery mechanism. Health communication should build on local nosologies rather than against them, using the very Fongbe terms patients use so that biomedical messages attach to existing categories.

We stress that the effectiveness of traditional-healer collaboration for skin-NTD outcomes has not been demonstrated in Benin; it should be piloted and evaluated on measurable endpoints, such as time-to-diagnosis and referral completion, before scale-up, rather than assumed.

The framework sorts barriers by where they concentrate; it does not imply that each must be tackled by a separate, single-purpose activity. In practice an integrated package, combining strengthened community structures for detection and stigma reduction with decentralized, affordable services, can act simultaneously on a shared barrier (cost) and on locally concentrated ones (access and infrastructure); the framework indicates which components should be applied uniformly and which should be intensified in specific districts. We did not measure the return on investment of such integrated delivery, and its effectiveness and cost-effectiveness for skin NTDs remain to be demonstrated; quantifying incremental cost per additional case detected and per unit of delay or stigma averted is a priority for the validation programme outlined in Section 5.3.

From a cost and feasibility perspective, the two intervention logics are not equivalent in their budgetary implications. Removing point-of-care costs is a recurrent expense that scales with case load and is therefore predictable but politically dependent on stable financial-protection mechanisms; an early step that requires no new infrastructure would be the integration of skin-NTD diagnosis and treatment into the existing ARCH benefit package, with marginal cost low enough to absorb through existing administrative channels. By contrast, targeted infrastructural investment in remote communes (decentralized detection points, transport support, peripheral staff strengthening) is a capital expense with high up-front costs but long-term efficiency gains through earlier presentation and shorter hospitalization. Prioritization should therefore distinguish the two: financial-protection measures can be advanced rapidly within current envelopes, whereas infrastructural investment requires dedicated allocations from the 10.13039/501100004726Ministry of Health and complementary support from technical partners. For policy makers, the implication is operational rather than diagnostic. The Ministry of Health and the National Programme for the Control of Leprosy, Buruli Ulcer and Yaws can use this classification to argue, district by district, for differentiated funding envelopes. Technical partners such as the World Health Organization, the Anesvad Foundation and the Raoul Follereau Foundation can integrate the same classification into their planning, allocating their budgets toward the cells where their comparative advantage is greatest (shared interventions for normative work and pooled financing; targeted investment for site-specific infrastructure and outreach). In all cases, the tool is presented as a decision aid, not as an algorithm that pre-empts the political negotiations through which programme priorities are ultimately set.

### 5.3 Strengths and limitations

Strengths include a sampling frame built from household enumeration rather than from CDTUB registers alone, combined with interviewing away from health facilities, which reduces over-representation of patients already linked to biomedical care; a multidimensional adjustment for socioeconomic position rather than reliance on a single composite; and a robustness check across three different specifications of socioeconomic adjustment, with concordant results.

Several limitations should be acknowledged. The cross-sectional design precludes causal inference. The comparison rests on only two communes, which limits transferability; consistent with case-based research, we do not claim statistical generalizability but describe both settings in detail so that readers can assess applicability elsewhere. Although the community-based interviewing strategy reduces over-representation of CDTUB-linked patients, the frame was nonetheless drawn from CDTUB catchment areas. Three populations are therefore likely under-represented: persons affected by a skin-NTD living in villages entirely outside any CDTUB outreach radius; persons who never sought care of any kind and remained invisible to the CDTUB register and to community enumerators; and persons whose lesions had healed or were attributed to non-NTD causes at the time of enumeration. By construction, these populations are likely to face barriers at least as severe as those reported here. The estimates of barrier burden should therefore be read as conservative, and the between-commune contrasts as a lower bound on the true structural differential. Disease knowledge was measured by recognition from a list rather than free recall and over-states genuine knowledge. Household membership was not recorded, so we could not determine whether several participants were drawn from the same household, nor adjust for any resulting clustering; the confidence intervals reported here may therefore be slightly too narrow. Recruitment was consecutive rather than random within the enumerated frame, so households and individuals that were easier to reach are likely to be over-represented and the percentages reported here should not be read as population prevalences. Because the number of individuals approached and the number declining were not recorded, we cannot assess whether those who declined differed from those who took part. Three participants were aged under 18; written parental consent and oral assent were obtained for each, but the study documentation did not include an age-adapted assent form, which should be anticipated in future protocols. Zagnanado, with approximately 55,000 inhabitants against 126,000 in Allada, contributed 45.4% of the sample, so figures pooled across the two communes are unweighted sample means rather than estimates for the two communes combined. Urbanicity of residence was self-reported without an operational definition, which limits its interpretation as an adjustment variable across the two communes. We did not apply a formal correction for multiple comparisons, and reporting only the effects significant at *P* < 0.001 does not resolve this. With three outcomes and five covariates tested in each model, the nominal *P* values overstate the evidence against the null, and a threshold chosen after inspecting the results does not restore their interpretation. The regression results are therefore best read as exploratory: the two large structural associations are findings to be confirmed in an independent sample rather than confirmatory tests, and the smaller covariate effects should not be interpreted individually. The absence of a detectable commune effect on treatment cost should be read as failure to detect a differential rather than as proof of equivalence; with the observed sample size, modest differentials might have escaped detection. Finally, the framework is illustrated on two sites and has not been validated as an instrument. A meaningful validation programme should proceed in three stages: replication of the burden and variability assessment across additional endemic districts of Benin (a minimum of four to six sites is desirable for credible discrimination of the four cells), followed by extension to other West African endemic contexts; consultation of national NTD programme managers and operational partners to assess face validity and decision-making utility; and an embedded evaluation in which the tool guides actual resource-allocation choices in a pilot district, with monitoring of effective coverage as the outcome. Inter-rater reliability was not quantified with a kappa coefficient, as the coders' independent code assignments were not retained; qualitative rigour rested instead on double coding with consensus resolution and on triangulation across respondent groups.

## 6 Conclusion

Skin-NTDs in Zagnanado and Allada affect economically active rural adults with low education and precarious incomes; 91.3% confirmed persistence. Treatment cost was a high-burden barrier in both communes, with no detectable commune effect after adjustment, consistent with operation as a shared constraint. Structural barriers (limited access and lack of infrastructure) were concentrated in the more isolated commune and remained so after multidimensional adjustment for socioeconomic position, robust across three different specifications. This pattern suggests that programme implementation should be tailored to the type of barrier rather than uniformly designed: shared interventions for shared barriers, targeted investment for locally concentrated ones. We offer a preliminary framework that operationalizes this distinction (Fig. 2). It was developed from two purposively selected communes and has not been externally validated. Whether it transfers to other districts or countries is an open question rather than a property we have demonstrated, and answering it should precede, not follow, any use of the framework in actual resource-allocation decisions.

## Declaration of generative AI in the writing process.

During the preparation of this manuscript, the authors used an AI-assisted tool to improve the language and readability of the text. The authors reviewed and edited the content and take full responsibility for the content of the publication.

## CRediT authorship contribution statement

**Roch Houngnihin:** Supervision, Methodology, Conceptualization. **Mélissa Biao:** Writing – review & editing, Writing – original draft, Project administration, Investigation, Formal analysis. **Bernice Gbèbioho:** Writing – review & editing, Formal analysis. **Fréjus Togonou:** Writing – review & editing, Formal analysis. **Daniel Azonchiga:** Writing – review & editing, Formal analysis.

## Funding

This research was funded by the Anesvad Foundation, the Fondation Raoul Follereau and the Dioraphte Foundation within the DESABEN-NTD project. The funders had no role in study design, data collection, analysis, interpretation or manuscript preparation.

## Declaration of competing interest

The authors declare that they have no known competing financial interests or personal relationships that could have appeared to influence the work reported in this paper.

## Data Availability

The datasets generated and analyzed during the current study are available from the corresponding author on reasonable request, subject to a data-use agreement to protect participant confidentiality. Replication code for the statistical analyses is provided as supplementary material. The following are the supplementary data related to this article.

**Fig. S1:**
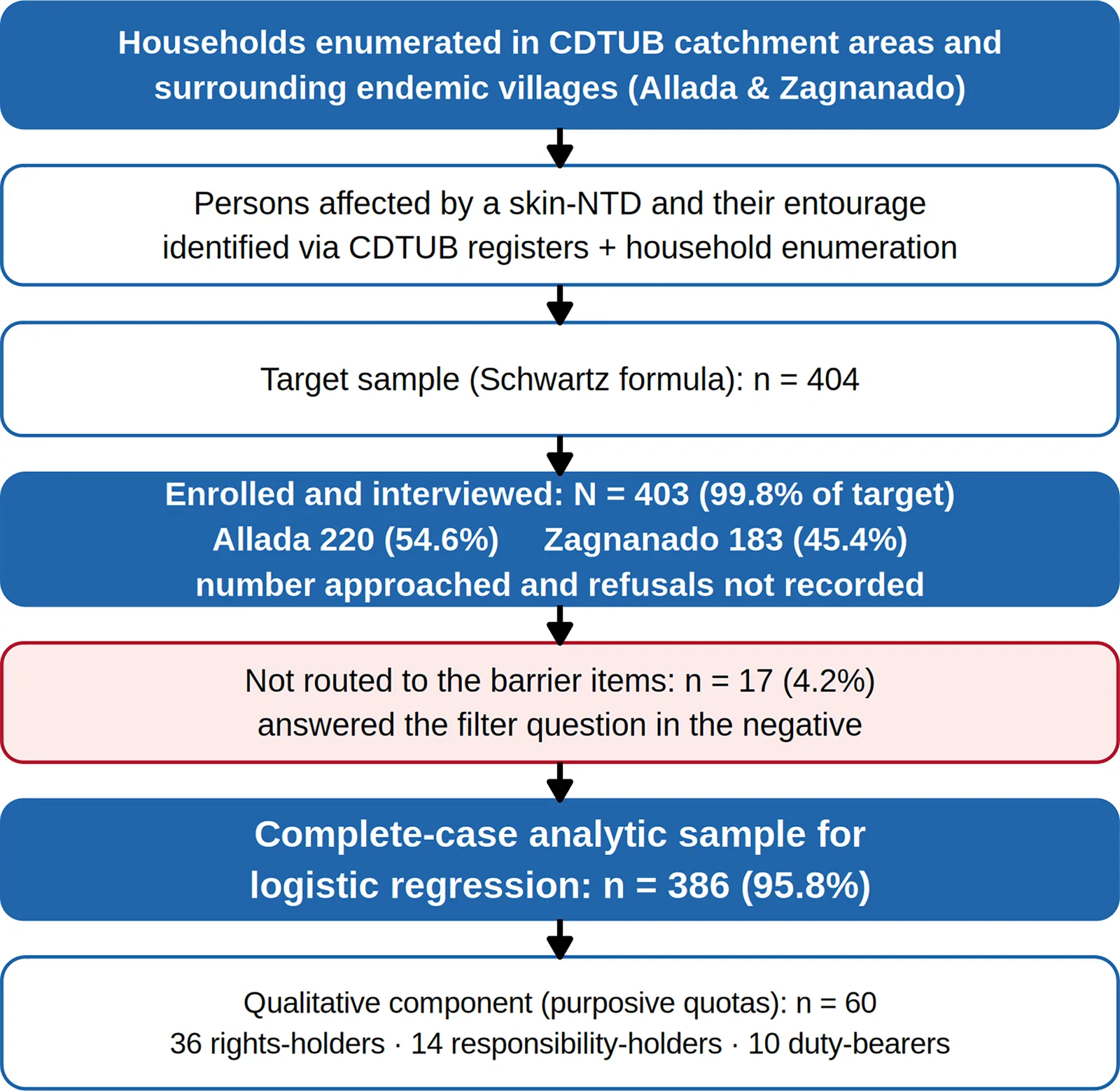
STROBE participant-flow diagram. From household enumeration in CDTUB catchment areas to the enrolled sample (*N* = 403), the complete-case analytic sample for regression (*n* = 386, after excluding the 17 participants not routed to the barrier items) and the purposive qualitative sample (*n* = 60); Source: DESABEN-NTD, 2025.

Supplementary material 1 STROBE participant-flow diagram. From household enumeration in CDTUB catchment areas to the enrolled sample (*N* = 403), the complete-case analytic sample for regression (*n* = 386, after excluding the 17 participants not routed to the barrier items) and the purposive qualitative sample (*n* = 60); Source: DESABEN-NTD, 2025. Supplementary material 1 Supplementary data to this article can be found online at https://doi.org/10.1016/j.dialog.2026.100350.
