# Supplementary material for: Shared and targeted barriers to care for skin-related neglected tropical diseases in two endemic communes of Benin: Toward a framework for resource allocation
Source: Dialogues Health. 2026 Sep 16;9:100350. doi: 10.1016/j.dialog.2026.100350 (PMC13659760; doi:10.1016/j.dialog.2026.100350)
Supplement: Supplementary file 1 [file mmc2.docx]

**Supplementary material**

**Table S2a.** *Missingness on barrier outcomes by commune.*

| **Commune** | **Complete (n)** | **Missing (n)** | **Total** |
| --- | --- | --- | --- |
| Allada | 209 | 11 | 220 |
| Zagnanado | 177 | 6 | 183 |
| Total | 386 | 17 | 403 |

*Note. Pearson χ² = 0.37, df = 1, P = 0.54: missingness did not differ between communes.*

**Table S2b.** *Comparison of included (n = 386) and excluded (n = 17) participants.*

| **Variable** | **Test** | **P** |
| --- | --- | --- |
| Sex | χ² | 0.96 |
| Education | χ² | 0.018 |
| Urbanicity | χ² | 0.88 |
| Household size | χ² | 0.85 |
| Occupation | χ² | 0.42 |
| Age (mean 36.5 vs 38.9) | t-test | 0.38 |

*Note. Included and excluded participants were similar on all variables except education (P = 0.018); given the small number excluded (4.2%), complete-case analysis is unlikely to bias the estimates.*

**Table S3.** *Multivariable logistic regression, full model output (n = 386). (a) Limited access to care.*

| **Predictor** | **aOR** | **95% CI** | **P** |
| --- | --- | --- | --- |
| Commune (Zagnanado vs Allada) | 8.09 | 4.72-13.86 | <0.001 |
| Sex (male vs female) | 0.78 | 0.46-1.33 | 0.363 |
| Education (per level) | 0.80 | 0.59-1.08 | 0.146 |
| Urbanicity (per level) | 1.04 | 0.71-1.52 | 0.833 |
| Household size (per level) | 0.89 | 0.70-1.13 | 0.324 |
| Occupation: artisan/trader | 0.89 | 0.46-1.72 | 0.722 |
| Occupation: manual worker | 1.87 | 0.52-6.76 | 0.341 |
| Occupation: professional | 7.41 | 2.60-21.16 | <0.001 |
| Occupation: unemployed | 1.11 | 0.35-3.54 | 0.854 |
| Occupation: other | 1.58 | 0.69-3.61 | 0.275 |

**Table S3 (continued).** *Multivariable logistic regression, full model output (n = 386). (b) Lack of infrastructure.*

| **Predictor** | **aOR** | **95% CI** | **P** |
| --- | --- | --- | --- |
| Commune (Zagnanado vs Allada) | 12.91 | 6.71-24.81 | <0.001 |
| Sex (male vs female) | 1.44 | 0.80-2.59 | 0.220 |
| Education (per level) | 1.02 | 0.73-1.42 | 0.898 |
| Urbanicity (per level) | 1.14 | 0.76-1.71 | 0.511 |
| Household size (per level) | 1.27 | 0.97-1.66 | 0.081 |
| Occupation: artisan/trader | 1.26 | 0.62-2.59 | 0.526 |
| Occupation: manual worker | 0.38 | 0.09-1.53 | 0.172 |
| Occupation: professional | 2.75 | 0.81-9.27 | 0.104 |
| Occupation: unemployed | 1.20 | 0.38-3.86 | 0.755 |
| Occupation: other | 0.84 | 0.32-2.20 | 0.716 |

**Table S3 (continued).** *Multivariable logistic regression, full model output (n = 386). (c) Treatment cost.*

| **Predictor** | **aOR** | **95% CI** | **P** |
| --- | --- | --- | --- |
| Commune (Zagnanado vs Allada) | 0.72 | 0.35-1.50 | 0.380 |
| Sex (male vs female) | 0.99 | 0.48-2.05 | 0.988 |
| Education (per level) | 0.82 | 0.55-1.21 | 0.315 |
| Urbanicity (per level) | 0.63 | 0.38-1.05 | 0.076 |
| Household size (per level) | 1.18 | 0.84-1.66 | 0.346 |
| Occupation: artisan/trader | 1.22 | 0.49-3.03 | 0.663 |
| Occupation: manual worker | 3.93 | 0.43-35.51 | 0.224 |
| Occupation: professional | 1.87 | 0.40-8.76 | 0.426 |
| Occupation: unemployed | 2.79 | 0.31-24.71 | 0.357 |
| Occupation: other | 1.10 | 0.38-3.24 | 0.864 |

*Note. All three models additionally adjust for occupation (farmer/fisher as reference). Reference categories: Allada and female.*

**Table S4.** *Between-commune effect (Zagnanado vs Allada) under three specifications of socioeconomic adjustment.*

| **Outcome** | **Principal model** | **Composite SES (PCA)** | **Field SES (3-level)** |
| --- | --- | --- | --- |
| Limited access | 8.09 (4.72-13.86) | 5.72 (3.71-8.95) | 5.83 (3.75-9.19) |
| Lack of infrastructure | 12.91 (6.71-24.81) | 10.38 (6.19-18.08) | 10.74 (6.37-18.78) |
| Treatment cost | 0.72 (0.35-1.50) | 0.68 (0.36-1.27) | 0.68 (0.36-1.28) |

*Note. aOR (95% CI). PCA composite from education, urbanicity and inverted household size (PC1 loadings 0.71, 0.61, 0.36; variance explained 42.4%). The structural-barrier contrasts remain large and highly significant across all three specifications (all P < 0.001), and treatment cost remains non-significant.*

**Table S5.** *Sensitivity of the quadrant classification to the burden cut-off.*

| **Barrier** | **% Allada / Zagn.** | **40%** | **50%** | **60%** | **Classification** |
| --- | --- | --- | --- | --- | --- |
| Treatment cost | 85.9 / 83.1 | High/High | High/High | High/High | Shared (unchanged) |
| Limited access | 26.4 / 66.7 | Low/High | Low/High | Low/High | Targeted (unchanged) |
| Lack of infrastructure | 10.0 / 53.0 | Low/High | Low/High | Low/High | Targeted (unchanged) |

*Note. The classification is invariant across cut-offs of 40-60%.*

**Table 1** *Socio-demographic characteristics by commune.*

| **Characteristic** | **Allada (n=220)** | **Zagnanado (n=183)** | **Total (n=403)** | **P** |
| --- | --- | --- | --- | --- |
| Sex: female | 111 (50.5) | 93 (50.8) | 204 (50.6) | 1.00 |
| Sex: male | 109 (49.5) | 90 (49.2) | 199 (49.4) |  |
| Age <25 | 13 (5.9) | 26 (14.2) | 39 (9.7) | 0.019 |
| Age 25-49 | 176 (80.0) | 134 (73.2) | 310 (76.9) |  |
| Age >49 | 31 (14.1) | 23 (12.6) | 54 (13.4) |  |
| Christian | 139 (63.2) | 110 (60.1) | 249 (61.8) | 0.002 |
| Endogenous | 67 (30.5) | 52 (28.4) | 119 (29.5) |  |
| Muslim | 6 (2.7) | 0 (0.0) | 6 (1.5) |  |
| Not stated | 8 (3.6) | 21 (11.5) | 29 (7.2) |  |
| Education: none | 73 (33.2) | 82 (44.8) | 155 (38.5) | 0.013 |
| Primary | 57 (25.9) | 54 (29.5) | 111 (27.5) |  |
| Secondary | 79 (35.9) | 41 (22.4) | 120 (29.8) |  |
| University | 11 (5.0) | 6 (3.3) | 17 (4.2) |  |
| Residence: rural | 125 (56.8) | 77 (42.1) | 202 (50.1) | <0.001 |
| Semi-urban | 89 (40.5) | 70 (38.3) | 159 (39.5) |  |
| Urban | 6 (2.7) | 36 (19.7) | 42 (10.4) |  |

*Note. n (%). Pearson χ² (Fisher where expected counts < 5).*

**Table 5 (revised).** *NTDs recognised from a predefined list, by commune.*

| **NTD** | **Allada n (%)** | **Zagnanado n (%)** | **P** |
| --- | --- | --- | --- |
| Lymphatic filariasis | 125 (56.8) | 90 (49.2) | 0.153 |
| Onchocerciasis | 124 (56.4) | 51 (27.9) | <0.001 |
| Sleeping sickness | 109 (49.5) | 57 (31.1) | <0.001 |
| Leishmaniasis | 55 (25.0) | 57 (31.1) | 0.208 |
| Leprosy / mycobacterioses | 6 (2.7) | 64 (35.0) | <0.001 |
| Chagas disease | 7 (3.2) | 69 (37.7) | <0.001 |
| Buruli ulcer | 14 (6.4) | 15 (8.2) | 0.606 |
| Other | 76 (34.5) | 4 (2.2) | <0.001 |

*Note. n (%) within commune. The leprosy row corresponds to the dataset’s ‘non-tuberculous mycobacterioses’ field (n = 70), the reproducible value from the study data.*
